# Arylcoumarin perturbs SARS-CoV-2 pathogenesis by targeting the S-protein/ACE2 interaction

**DOI:** 10.1038/s41598-022-20759-7

**Published:** 2022-10-11

**Authors:** Ruhar Singh, Abhijeet Kumar, Jitendra Subhash Rane, Rajni Khan, Garima Tripathi, Amrendra K. Ajay, Amresh Prakash, Shashikant Ray

**Affiliations:** 1 School of Computational and Integrative Sciences, Jawaharlal Nehru University, New Delhi, India; 2 Department of Chemistry, Mahatma Gandhi Central University, Motihari, 845401 India; 3 Department of Biosciences and Bioengineering, Indian Institute of Technology Bombay, Mumbai, 400076 India; 4 Department of Pharmacology and Toxicology, National Institute of Pharmaceutical Education and Research, Hajipur, 844102 India; 5 Department of Chemistry, T.N.B. College, Bhagalpur, Tilka Manjhi Bhagalpur University, Bhagalpur, 812007 India; 6 Renal Division, Department of Medicine, Brigham and Women’s Hospital and Harvard Medical School, Boston, MA 02115 USA; 7 Amity Institute of Integrative Sciences and Health, Amity University Haryana, Gurgaon, 122413 India; 8 Department of Biotechnology, Mahatma Gandhi Central University, Motihari, 845401 India

**Keywords:** Computational biology and bioinformatics, Drug discovery

## Abstract

The vaccination drive against COVID-19 worldwide was quite successful. However, the second wave of infections was even more disastrous. There was a rapid increase in reinfections and human deaths due to the appearance of new SARS-CoV-2 variants. The viral genome mutations in the variants were acquired while passing through different human hosts that could escape antibodies in convalescent or vaccinated individuals. The treatment was based on oxygen supplements and supportive protocols due to the lack of a specific drug. In this study, we identified three lead inhibitors of arylated coumarin derivatives 4,6,8-tri(naphthalen-2-yl)-2H-chromen-2-one (NF1), 8-(4-hydroxyphenyl)-4,6-di(naphthalen-2-yl)-2H-chromen-2-one (NF12) and 8-(4-hydroxyphenyl)-3,6-di(naphthalen-2-yl)-2H-chromen-2-one (NF-13) that showed higher binding affinity towards the junction of SARS-CoV-2 spike glycoprotein (S-protein) and human angiotensin-converting enzyme 2 (ACE2) receptor. Using molecular docking analysis, we identified the putative binding sites of these potent inhibitors. Notably, molecular dynamics (MD) simulation and MM-PBSA studies confirmed that these inhibitors have the potential ability to bind Spike-protein/ACE2 protein complex with minimal energy. Further, the two major concerns are an adaptive mutation of spike proteins- N501Y and D614G which displayed strong affinity towards NF-13 in docking analysis. Additionally, in vitro and in vivo studies are required to confirm the above findings and develop the inhibitors as potential drugs against SARS-CoV-2.

## Introduction

The World Health Organization (WHO) declared the SARS-CoV-2 infection as an epidemic and public health emergency in January 2020 and named it COVID-19, which rapidly spread into the entire world approximately within a few months that not only threatened public health across the globe but also drastically increased the human mortality^1–4^. SARS-CoV-2 belongs to the Coronavirus family-β-coronavirus 2B lineage, a novel coronavirus that infects humans^4–8^. The initial genome sequencing studies showed 96% identity similarity with the bat SARS-like coronavirus strain BatCov RaTG13^6,9–12^. The positive-sense RNA genome contains 14 open reading frames (ORFs) that codes for 16 non-structural proteins which form the replicase complex, four structural proteins- spike (S), envelope (E), membrane (M), and nucleocapsid (N), and nine accessory proteins^7,13–15^. The entry into the host cell is mediated by the spike protein, which has a receptor-binding domain (RBD) that mediates attachment with the cellular receptor human angiotensin-converting enzyme 2 (hACE2)^16–19^. The various vaccines designed, based on the disease pathogenesis studies, are being administered worldwide in humans as a preventive measure; however, none of the candidate vaccines promises 100% efficacy to contain the disease^20–22^. Hence, many reinfection cases have surfaced even after two doses of vaccination, and still, there is no standard line treatment for those already suffering from the disease^23^. Reinfections are attributed to the appearance of new mutant strains of SARS-CoV-2 B.1.1.7 in the UK, 501Y.V2 in South Africa, and B1.617 in India, which can escape the antibodies in vaccinated or convalescent persons^24–26^. The new strains are deadlier, with even faster transmission among humans raising the human mortality rates due to the disease^26^.

Studies showed that N501Y mutation is crucial in SARS-CoV-2 variants for the higher rate of transmission which may be due to the introduction of additional π–π and π–cation interactions in RBD-ACE2 complexes, as confirmed by molecular dynamic simulation studies^27^. N501Y mutation improves the viral spike protein affinity for cellular receptors, which facilitates the enhanced viral transmission in SARS-CoV-2 variants^28^. The functional implication of N501Y was more confirmed as it was found to be the only common mutation between the variants Alpha (B.1.1.7, 501Y.V1), Beta (B.1.351, 501Y.V2), and Gamma (P.1, 501Y.V3), in the RBD of S glycoprotein^29^. Further, N501Y mutation in the spike protein is reported to modify the interaction between hACE2 and Human- derived Antibody^30^. D164G mutation in the S-protein is also responsible for the enhanced infectivity of the SARS-CoV-2 variants^31^. GISAID data showed that the increase in the frequency of the D164G mutation variant globally, proved it as a dominant form in the pandemic. Clinical data, as well as experimental data showed, that D164G variants are associated with increased viral load and viral infectivity among the human populations^32^. Although the mutation did not enhance the severity of the disease^33^. Studies also showed an increase in the S- protein assemblage in the virion due to the DG164G mutation^34^.

Initially, to control the disease, various research groups tried to repurpose existing drugs. The SARS-CoV-2 was genetically sequenced in January 2020, which led to the escalated rate of drug targets screening against COVID-19 by scientific groups; however, there is no specific effective drug available to combat it^35,36^. Besides, the novel coronavirus has developed mechanisms to escape the antibodies generated in individuals after vaccination or prior COVID infections^37^. The titer value of antibodies generated does not reach the required optimum level to counteract the viral antigens; as a result, there is a failure in controlling the disease^37^. Hence, the development of a specific drug will strengthen the second line of defense mechanism and help treat COVID-19 infected patients^38,39^. The pathogenesis of SARS-CoV-2 starts with the interaction from the ACE2 receptor located in the lungs, heart, kidney, small intestines, and gallbladders, etc.^40,41^. Therefore the inhibitor which interrupts the interaction between the SARS-CoV2-S protein and ACE2 (S-protein-ACE2) receptor could potentially inhibit the infection in the host cells^8,42,43^. Currently, different phytochemical derivatives have been explored to find out the potential inhibitor against SARS-CoV-2.

Compounds with coumarin scaffolds exhibit a diverse range of biological activities such as anti-bacterial, anti-cancerous, anti-inflammatory, anti-HCV, anti-HIV, anti-leishmanial, anti-coagulant, etc.^44–46^. Coumarin is a naturally occurring compound that possesses drug-likeness properties such as stability, solubility, and low toxicity^47^. It has been reported that coumarin derivatives have antiviral properties against a broad range of viruses like HIV, Influenza, dengue virus, chikungunya virus, Enterovirus 71 (EV71), and coxsackie virus A16 (CVA16)^48^. Coumarin derivatives are known to target the proteins involved in the viral entry into the host cell, replication, transcription, and translation machinery, which are essential for the life cycle of viruses^48^. In addition, coumarin derivatives are also found to regulate the critical host signaling pathways like nuclear factor kappa-light-chain-enhancer of activated B cells (NF-κB) and inflammatory redox-sensitive pathways, thereby blocking the virus replication^48–50^. Further, coumarin derivatives eleutheroside B1 were found to inhibit the influenza virus replication by targeting the expression of the viral polymerase^51,52^. In addition, thiazolyl-coumarin hybrids were found to show promising antiviral properties against the H3N2 and H1N1 by targeting the protein neuraminidase, which is required for the formation of the viral envelope^48^.

The present study aims to explore the therapeutic potential of newly designed arylated coumarins, which could easily be accessed by employing synthetic simple reaction protocols such as cross-coupling arylation reaction and other conventional methods against SARS-CoV-2. We performed molecular docking and MD simulation to find out potent inhibitors against the S-protein-ACE2 protein complex from a library of 21 arylated coumarin derivatives. We found that NF1, NF12, and NF13 arylated coumarin derivatives interact at the junction of the S-protein-ACE2 protein complex with minimal energy. The molecular docking analysis revealed that the NF1, NF12, and NF13 interact at the interface of the S-protein-ACE2 protein complex with a binding energy of -12.1, -10.1 and -10.3 kcal/mol, respectively. In addition, we have also identified the putative binding site of the S-protein-ACE2 protein complex with NF1, NF12, and NF13 that can resist the novel coronavirus from entering the host cells. In addition, the molecular dynamics (MD) simulation study complemented the data of stronger binding affinity of NF1, NF12, and NF13 towards the interface of the S-protein-ACE2 protein complex.

## Material and methods

### Virtual screening of compounds

Structure based virtual screening for the library of 21 arylated coumarin derivatives compounds (Supplementary Table S1) was performed using PyRx^53^. Active site dimensions were set as grid size of center X = 90.27 Å, center Y = − 1.92 Å, center Z = 165.77 Å. The exhaustiveness was kept at 8 for the docking of each ligand. The docking was performed by vina wizard and top three ligands with minimum binding energy were chosen for further study.

### Protein preparation

Three-dimensional (3D) atomic coordinates of ACE2 in the bound state with SARS-CoV-2 chimeric receptor-binding domain (PDB ID: 6VW1) determined by X-ray crystallography with 2.68 Å resolution were retrieved from RCSB protein data bank (http://www.rcsb.org). All the co-crystalline water molecules and hetero atoms were removed, polar hydrogen atoms were added, and partial charges were assigned to the receptor. Finally, the receptor was saved in the PDBQT format.

### Ligand modeling

The compounds were sketched using ChemDraw and saved in the SDF file format. The SD files were converted to their corresponding three-dimensional (3D) structures and saved as .pdb format using PyMol^54^. The Gasteiger charges and non-polar hydrogens were assigned to the compounds using Autodock vina, and the compounds were saved in the PDBQT format.

### Molecular docking experiment

To investigate the binding poses, orientation, and binding affinity, molecular docking was conducted using the Autodock vina^55^. Both the ligand and receptor were converted into PDBQT format, which stores the atomic coordinates, partial charges, and Autodock vina atom types (AD4). First, the ligand and binding pocket were covered with the grid box with the dimension of 62 Å X 60 Å X 66 Å, and grid centers were kept as x = 15.23 Å, y =  − 10.57 Å, z = 17.25 Å. The docking simulation was then run at an exhaustiveness of 8 and set to only output the lowest energy pose, with search spacing 1 Å^55^. The molecular interactions of docked complexes were visualized using PyMol^54^. In the similar experimental condition spike protein mutants N501Y and D614G were docked with NF1, NF2 and NF3.

### MD simulation

MD simulations were performed for the ACE2-ligand complexes using AMBER-16 software, and the ligand unbound structure of ACE2 was used as a reference to calculate the protein-ligands dynamics and stability^56^. The AMBER ff99SB protein force field was used as parameters for the receptors, and the parameters for ligands were defined by force field GAFF and AM1-BCC charges, using antechamber^57–59^. The systems were prepared using tleap as described^60,61^. The prepared systems were placed in the center of a cubic simulation box with a 10 Å distance from the edge to reduce potential artifacts arising from periodicity and solvated with TIP3P water molecules. The counter ions (Na^+^Cl^−^) were added into the periodic box to neutralize the charge of the protein–ligand complex. Particle-Ewald summation evaluated long-range electrostatic interactions, 8 Å cut off used to compute vdW interactions, and PBC was defined for x,y, and z directions^62^. Bonded hydrogen atoms were constrained by applying the SHAKE algorithm. During the simulation, pressure and temperature were taken care of by Berendson’s barostat and Langevin thermostat, respectively^63,64^. Energy minimization of prepared systems was performed in three stages, each of 10,000 steps of steepest descent (SD) and conjugate gradient (CG) to relax the system. Further, each simulation system was gradually heated from 50 to 300 K in six steps, followed by 10,000 steps of SD and CG minimization, respectively. The systems were equilibrated by microcanonical (NVE) and NPT ensemble with collision frequency 2. Using the Amber tool pmemd.cuda, production runs were performed for 100 ns with a time step of 2 fs, and the trajectories were saved after every 10 ps. The obtained trajectories were analyzed through the cpptraj tool^65^.

### MD analysis

Using cpptraj, we analyzed the structural parameters, radius of gyration (Rg), root-mean-square deviation (RMSD), root-mean-square fluctuation (RMSF), solvent-accessible surface area (SASA), and hydrogen bond (H-bond) interactions. These properties were analyzed over time to determine the simulated system equilibration tendency. The H-bonds are calculated on the basis of geometric criteria with a cut-off distance of 3.0 Å between donor and acceptor, and the angle cutoff is 135°. The dimension reduction method principal component analysis (PCA) was calculated to define the conformational space occupied by protein during the simulation. And, applying the Boltzmann inversion with two PCs, PC1 and PC2, free-energy plots were calculated in the MATLAB platform^66,67^.

### Calculation of binding interactions using MM-PBSA and MM-GBSA

The binding free energy calculated using Molecular Mechanics Poisson-Boltzmann Surface Area (MM-PBSA) method implemented in AMBER 16 MD software package (https://ambermd.org/). The estimation of binding free energy using MM-PBSA involves molecular mechanics, continuum electrostatics, and solvent-accessible surface area. The binding free energy (ΔG_bind_) was calculated as the energy difference between the complex (G_complex_) and the receptor (G_protein_) and ligand (G_ligand_). The free energies were calculated from the ensemble generated from MD simulations using a program MMPBSA*.*py which is written in python. We carried out binding energy calculations involving both methods, MM-PBSA and MM-GBSA. Using this script, the calculated different energy components do not include the entropy contribution in the binding. Using the MM-PBSA approach, different energy components were calculated as,

1 $$\Delta {\text{G}}_{{{\text{bind}}}} = {\text{ G}}_{{{\text{complex}}}} - {\text{G}}_{{{\text{protein}}}} - {\text{G}}_{{{\text{ligand}}}} = \Delta {\text{E}}_{{{\text{MM}}}} + \Delta {\text{G}}_{{{\text{sol}}}} - {\text{T}}\Delta {\text{S}}$$

2 $$\Delta {\text{E}}_{{{\text{MM}}}} = \Delta {\text{E}}_{{{\text{bonded}}}} + \Delta {\text{E}}_{{{\text{nonbonded}}}} = \Delta {\text{E}}_{{{\text{bonded}}}} + \, (\Delta {\text{E}}_{{{\text{vdW}}}} + \Delta {\text{E}}_{{{\text{ele}}}} )$$

3 $$\Delta {\text{G}}_{{{\text{sol}}}} = \Delta {\text{G}}_{{{\text{polar}}}} + \Delta {\text{G}}_{{{\text{nonpolar}}}}$$

Finally, the free binding energy (ΔG_bind_) of ligands was calculated as,

4 $$\Delta {\text{G}}_{{{\text{bind}}}} = \Delta {\text{G}}_{{{\text{vdW}}}} + \Delta {\text{G}}_{{{\text{ele}}}} + \Delta {\text{G}}_{{{\text{polar}}}} + \Delta {\text{G}}_{{{\text{nonpolar}}}}$$

Further, Molecular Mechanics Generalized Born Surface Area (MM-GBSA) energy approximation method was also used to determine the binding constants. Here, the molecular mechanics term ΔEMM is defined as for MM-PBSA; however, in Generalized-Born (GB) model, the solvation free energy ${\mathrm{G}}_{\mathrm{sol}}$ of a solute molecule is calculated as, G_sol_ = G_sol-GB_ + G_surf_. The G_sol-GB_ represents the polar or electrostatics, and the non-polar component defines G_surf_ considering the SASA of solute. Furthermore, it also allows the analysis of the contributions of active site residues in stabilizing the ligands in energy terms by free energy decomposition analysis.

## Result and discussion

The in silico approach of drug designing has now become a vital tool for screening novel and potential drug candidates from a library of chemical or natural derivatives^42,68^. It helps to design a moiety against the specific target that facilitates the identification of lead molecules^68^. Molecular docking analysis helps to identify the strength of interaction between ligand and the receptor along with the identification of interacting amino acid residues in the ligand-receptor complex^42^. In the SARS-CoV-2 infection, the accessibility of the virus inside the host cell determines the infectivity and pathogenicity^7,16^. The spike glycoprotein of the SARS-CoV-2 virus has two main globular domains, i.e., S1 and S2. The S1 domain helps in the attachment of the COVID-19 virus with the ACE2 receptor^69^.This is followed by the cleavage of spike protein at the junction of S1/S2 domains by the host cell proteases (TMPRSS211, a serine protease, and lysosomal proteases cathepsins), ensuing the conformation change in the S2 domain^7,70^. This results in the entry of the virus into the host cell, followed by pathogenicity^7^. Therefore, targeting the interface of the S-protein-ACE2 protein complex may help to identify a lead inhibitor for the development of anti-COVID-19 agents. Herein, we aimed to identify a lead molecule that may bind at the junction of the S-protein-ACE2 protein complex and thereby perturbing the interaction of the S-protein-ACE2 protein complex followed by the inhibition of viral pathogenesis.

### Molecular docking analysis

**Figure 1:**
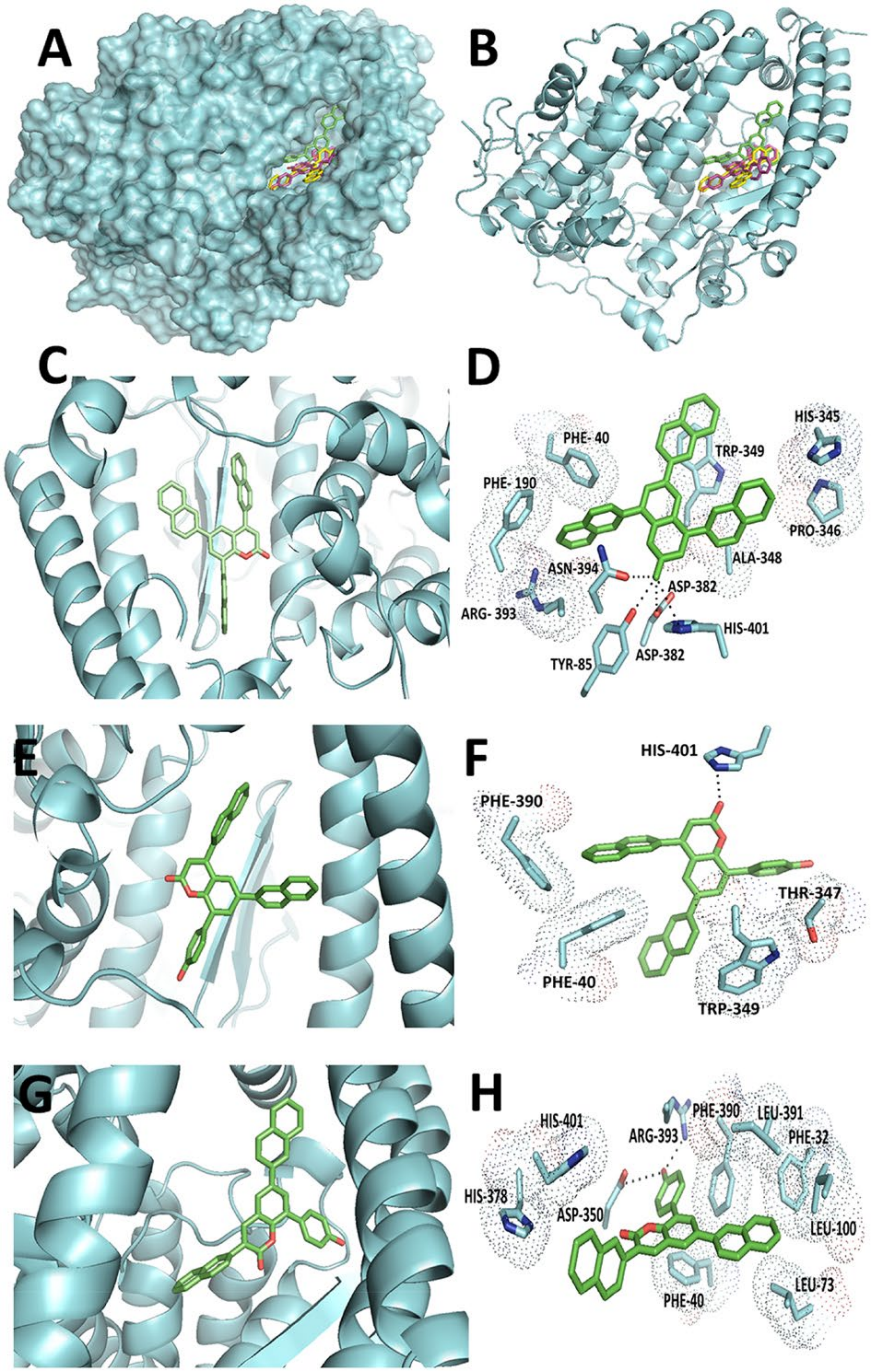
Binding site of NF-1, NF-12, and NF-13 on S-protein-ACE2 complex. (**A**) The binding site of NF-1, NF-12, and NF-13 on the S-protein-ACE2 receptor complex. (**B**) The putative binding site of NF-1, NF-12, and NF-13 on the S-protein-ACE2 receptor complex. (**C**) The zoomed view of the binding site of NF-1 on the S-protein-ACE2 receptor complex. (**D**) The interacting amino acid residues of ACE2 with NF-1. (**E**) The zoomed view of the binding site of NF-12 on the S-protein-ACE2 receptor complex. (**F**) The interacting amino acid residues of ACE2 with NF-12. (**G**) The zoomed view of the binding site of NF-13 on the S-protein-ACE2receptor complex. (H) The interacting amino acid residues of ACE2 with NF-13.

The docking studies revealed that all three triarylcoumarins, NF1, NF12, and NF13, bind to the ACE2 protein efficiently through H-bonding and non-covalent such as π–π, C–H..π interactions (Fig. 1A,B). Due to the presence of three aryl groups that add hydrophobicity, these triarylcoumarins prefer to interact mainly with the hydrophobic amino acids such as PHE, TRP, ALA, etc. (Table 1). The polar amino acids participate in the hydrogen bonding with the oxygen atom of the carbonyl group present in coumarin. As shown in Fig. 1C,D, the naphthyl group present at the 8th position of the coumarin ring of NF1 is interacting through some sort of π–π interactions with the phenyl rings of PHE40 and PHE390. Similar interactions were also observed between the indole ring of TRP349 and the naphthyl group present at the 6th position of the coumarin ring. The C–H..π interaction could be observed between one of the C–H of naphthyl rings present at the 4th position and between PRO346. The oxygen atom of the carbonyl group of coumarin ring is involved in hydrogen bonding with the hydroxyl group of ASN394, TYR385, ASP382, and with one of the N atoms of the imidazole ring of HIS401. Similarly, C–H..π interaction was found between the amino acid PHE-390 and 4-naphthyl ring of ligand NF12 (Fig. 1E,F). This ring was also involved in the π-π interaction with PHE 40. Here, HIS401 was also involved in the H-bonding with the carbonyl oxygen. However, in the case of THR347 (C–H..π) and TRP349 (π–π), hydrophobic interaction was there. The benzopyrone ring and phenyl ring present at 8th position of coumarin ring of NF13 ligand interact with PHE40 and PHE390 respectively through non-covalent π-π interaction (Fig. 1G,H). The polar amino acids, including ASP350 and ARG393 of ACE2, interact with the hydroxyl group of 4-hydroxyphenyl ring present at the 8th position of coumarin ring through hydrogen bonding. HIS401 also forms H-bonding with the oxygen present at carbonyl group of pyrone ring interaction of Leu73, Leu39, Leu100, and PHE32 are hydrophobic interactions. HIS401 also forms hydrophobic interaction.

**Table 1:** The Bold highlighted residues (ASN 394, TYR 385, ASP 382, HIS 401, ASP 350, ARG 393) show hydrophilic interaction while other residues are showing hydrophobic interaction.

Compounds	Docking score (kcal/mol)	Interacting residues of ACE2
NF1	− 12.1	PHE 40, PHE 390, ARG 393, **ASN 394, TYR 385, ASP 382, HIS 401**, ALA 348, TRP 349, PRO 346, HIS 345
NF12	− 10.1	PHE 40, PHE 390, ARG 393, **HIS 401**, PRO 346
NF13	− 10.3	HIS 401, HIS 378, **ASP 350, ARG 393**, PHE 390, LEU 391, PHE 40, LEU 73 LEU 100, PHE32

### Analysis from the MD simulation trajectories

**Figure 2:**
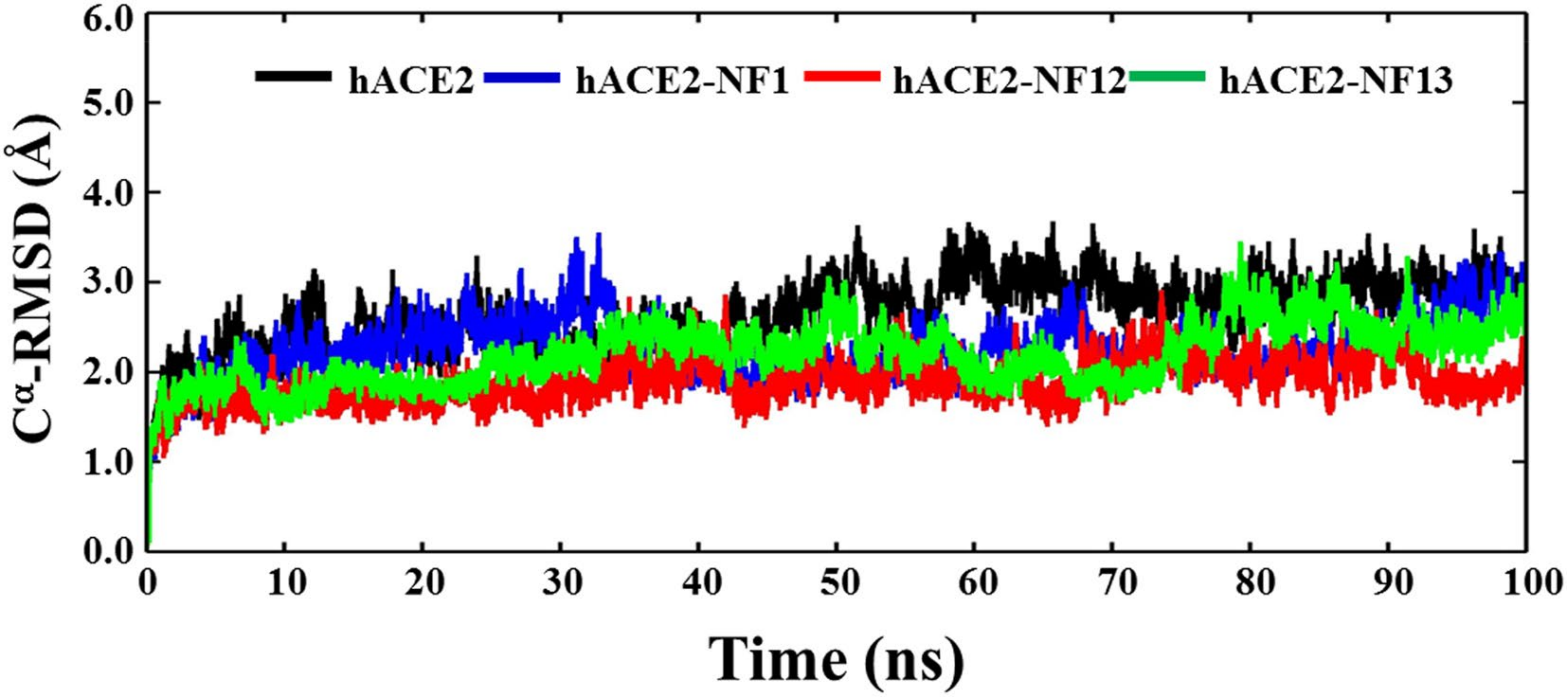
The backbone C^α^-RMSD plots reflecting the conformational stabilities of ACE2 (black) and docked complexes with ligands, NF1 (blue), NF12 (red), and NF13 (green). A similar color scheme is used to represent the plots for protein and protein–ligand complexes from Figs. 2, 3, 4, 5, 6 and 7.

**Figure 3:**
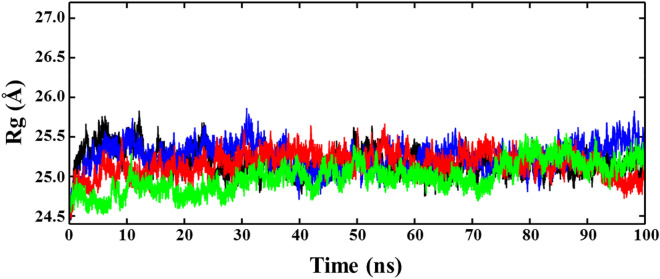
Time evolution plots of the radius of gyration (Rg) ofACE2 and protein–ligand complexes, ACE2-NF1, ACE2-NF12, and ACE2-NF13.

**Figure 4:**
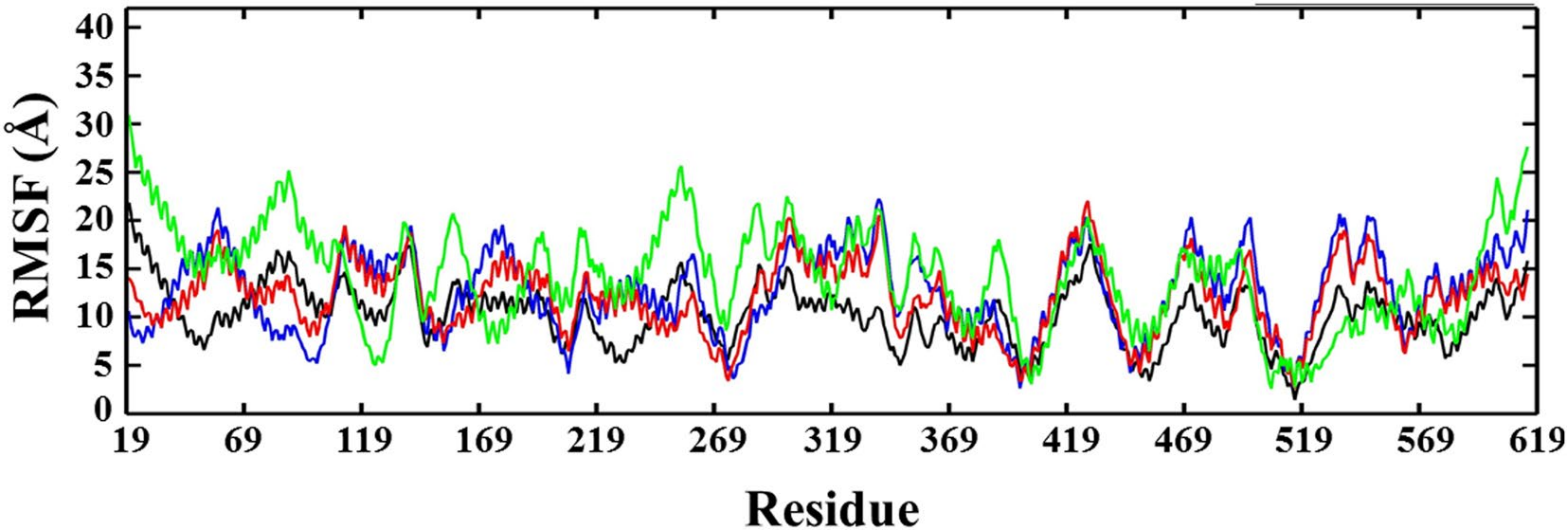
RMSF plots of ACE2 and docked complexes, ACE2-NF1, ACE2-NF12, and ACE2-NF13.

To identify the potential binding of promising docking hit ligands and the structural stability of protein–ligand complexes, we performed all atoms MD simulation in an aqueous environment for the period of 100 ns, at 300 K. Further, the MM-PBSA was explored to estimate the binding free energies of the ligands. At first, we computed the structural order parameters, RMSD **(**Fig. 2), Rg (Fig. 3), and SASA (Fig. 4), which provide a global idea about the conformational dynamics of the structure during the evolution of MD simulation time. The average values of RMSD, Rg, and SASA are enumerated in Table 2. Figure 2, the time evolution plots of Cα-RMSD show that the ligand unbound structure of ACE2 achieves equilibrium at ~ 20 ns, and it remained consistent up to 40 ns. A continuous increase in RMSD value of ~ 1.0–1.5 Å can be noticed during 40–60 ns; after that, a well-settled equilibrium was observed until the simulation ends at 100 ns. The average change in RMSD of ACE2 was noticed around 2.63 ± 0.40 Å, which was taken as a reference to compare the structural dynamics of ACE2-ligand complexes. The plot of ACE2-NF1 shows a continuous rise in RMSD up to the first 30 ns of simulation with the drifts of 0.5–1.5 Å. It achieves equilibrium gradually at ~ 40 ns and remains stable till 90 ns. However, the trajectory shows drifts at ~ 60–70 ns, and RMSD reach up to 3.0 Å at 90–100 ns indicates the less stable structural dynamics of NF1 binding with ACE2. The structure of ACE2-NF12 attains equilibrium quickly in the initial 5 ns and remains stable up to 60 ns. However, the slight deviation in trajectory was observed at ~ 65 ns that settled at ~ 70 ns, and the simulation ends with the average RMSD value of 1.89 ± 0.24 Å. The RMSD plot of ACE2-NF13 shows that it reaches equilibrium at ~ 10 ns and remains stable up to 50 ns. RMSD rises due to drifts at ~ 50–55 ns, which settles at ~ 60 ns. The further increase in RMSD can be seen at 70 ns, and the structural dynamics gradually shifted to RMSD 2.80 Å at ~ 80–100 ns.

**Table 2:** Average values of the structural order parameters (RMSD, Rg and SASA).

	RMSD (Å)	Rg (Å)	SASA (Å^2^)
ACE2	2.63 ± 0.40	25.17 ± 0.15	27,287.5 ± 395.27
ACE2-NF1	2.24 ± 0.34	25.24 ± 0.16	27,775.3 ± 476.57
ACE2-NF12	1.89 ± 0.24	25.15 ± 0.15	26,862.6 ± 350.41
ACE2-NF13	2.18 ± 0.35	24.99 ± 0.18	27,483.9 ± 480.17

The deviation in the structural compactness of ACE2 after and before the binding of ligands was determined by examining the radius of gyration (Rg) (Fig. 3). During the initial 20 ns, the Rg plot of ACE2 shows a deviation in structure due to the drifts of ~ 0.5–1.5 Å, which gradually settles at ~ 25 ns. After that, a continuous equilibrium of stable structure was observed during ~ 25–100 ns, having an average Rg value of 25.17 ± 0.15 Å. The Rg trajectory of ACE2-NF1 shows a consecutive sharp drift of 0.5–1.0 Å, during the initial 0–35 ns, which settled at 40 ns. After that, the conformational dynamics observed stable with an average Rg value of 25.24 ± 0.16 Å, but a slight deviation is noted in Rg at 90–100 ns. The Rg plot of ACE2-NF12 shows that it quickly attains equilibrium at ~ 5 ns, which remains consistent until the simulation finished at 100 ns, with an average Rg value of 25.15 ± 0.15 Å. The structure of ACE2-NF13 converges at ~ 10 ns and remains stable up to 70 ns. A gradual increase in Rg ~ 0.7 Å was seen at ~ 70 ns which settled at ~ 80 ns and the simulation ended with stable conformational dynamics of ACE2-NF13 with an average Rg value of 24.99 ± 0.18 Å.

Another structural analysis includes the solvent-accessible surface area (SASA), which provides an idea about the accessibility of solvent molecules in the structural stability of protein–ligand molecular interactions. Table 2 shows the marginal changes in SASA of ACE2 and ACE2-ligand complexes which signify that no conformational changes in protein upon binding with ligand molecules and ligand molecules were spatially well fitted at the binding site of ACE2 during the simulation time. The plots of SASA are shown in Supplementary Fig. S1.

To identify the local dynamics of amino acid residues involved in the conformational stability of ligand molecules, RMSF of all four systems at the residual level was calculated (Fig. 4). In this figure, we can see the less average fluctuation of ACE2-NF1 residues ranges Ala36-Trp69 and Ala348-Arg393 compared to the average atomic fluctuation of ACE2. The RMSF plot of ACE2-NF12 shows a remarkable change in the atomic fluctuation of N-terminal residues Phe32-Ser47 and Met62-Leu73, and the region around Ser105 and Ser106 suggesting the lower average fluctuation due to involve in interaction with ligand. The region around the Trp349 and Phe390 crucially formed the active site of ACE2, which was observed comparatively stable compared to ACE2. Furthermore, the lower peaks of hydrophobic residues around Tyr510 indicate the role of hydrophobic interactions in stabilizing the ligands at the active site of ACE2. Although the RMSF plot of ACE2-NF13 showed a higher average fluctuation of residues at N and C-terminal of proteins, but the reasonable drop in the average fluctuations of the ACE2 active site provides clear evidence of stable binding of NF13 with ACE2.

**Figure 5:**
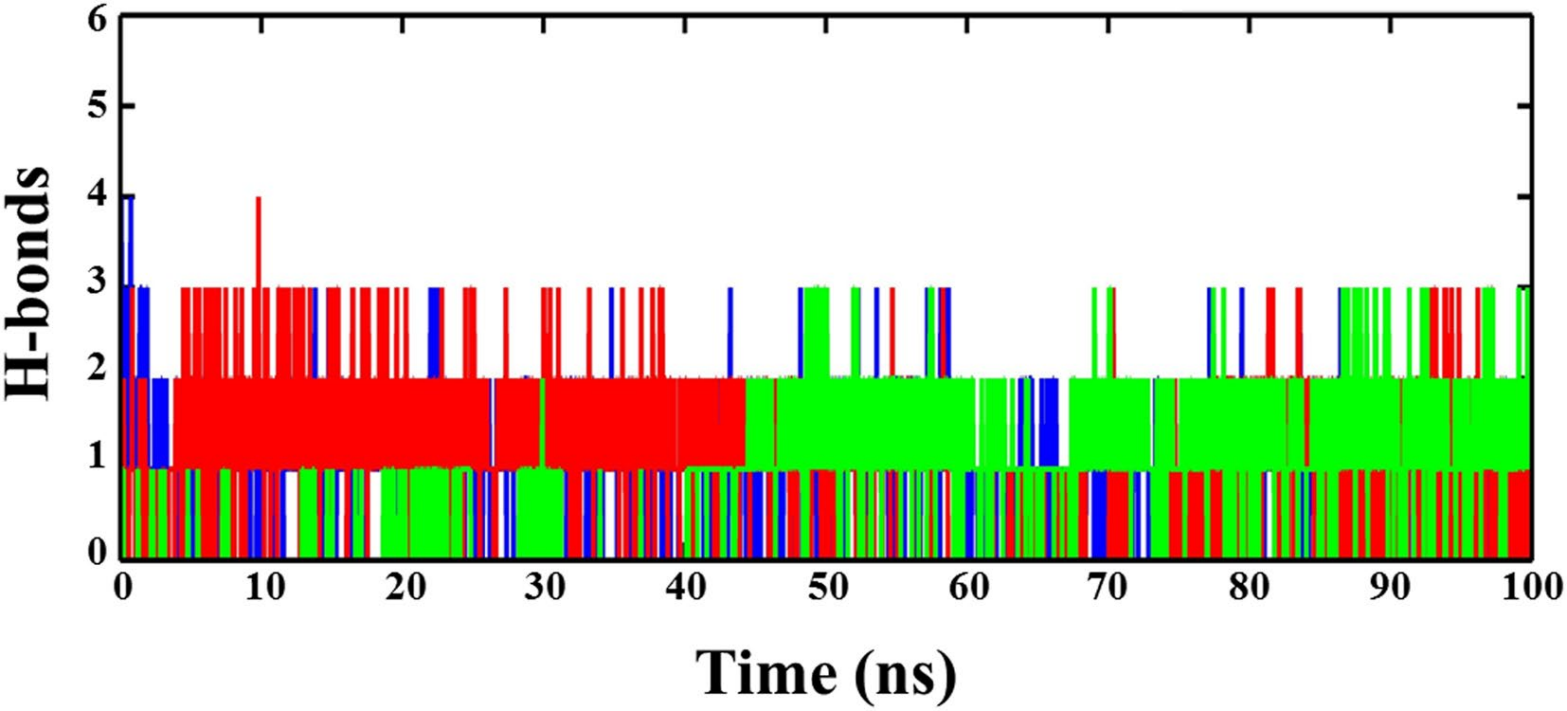
The time evolution plots of H-bond interactions between ACE2 with ligands NF1 (blue), NF12 (red), and NF13 (green).

Furthermore, we also measured the time evolution of hydrogen bonds (H-bonds) between ligands and ACE2 during the progression of simulation time (Fig. 5). Results show the maximum possibility of three H-bonds between ACE2 and NF1, out of which only two remained consistent during the simulation. Although the complex of ACE2-NF12 also shows the maximum occupancy of three H-bonds, but all the three H-bonds remain up to ~ 40 ns. One H-bond disappeared after 40 ns, and two H-bonds were lost at ~ 60 ns. Later on, all three H-bonds regained at ~ 80 ns, but only two H-bonds remained stable during the last 20 ns of simulation. Notably, the molecular interaction of NF13 with ACE2 shows the presence of only one H-bond up to ~ 40 ns. During the progression of simulation, three H-bonds appeared at ~ 30 ns which remained stable up to ~ 50 ns. However, during the last 50 ns of simulation, two H-bonds were observed consistently, and the third H-bond was appearing and disappearing intermittently. Thus, H-bond interactions suggest the stable binding of ligands at the active site of ACE2.

### Binding free energy

**Figure 6:**
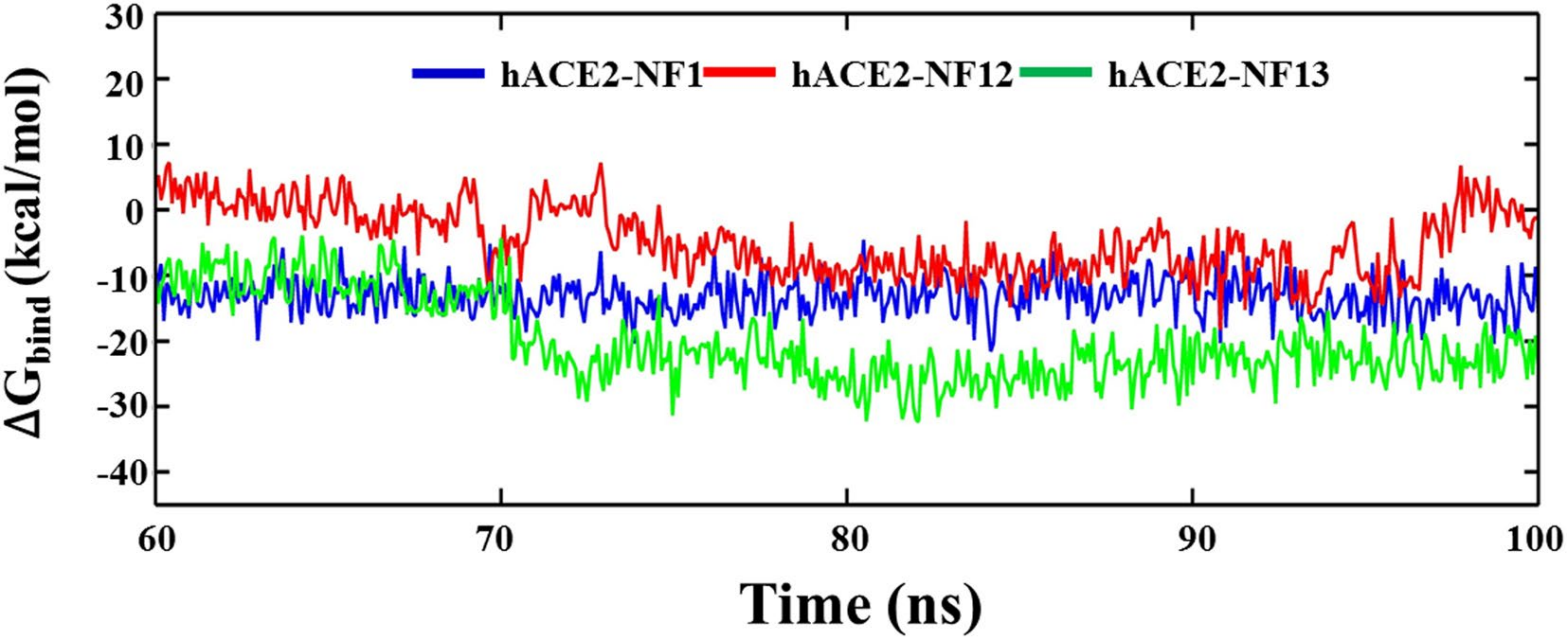
Binding free energy (ΔG_bind_) estimation of ligands, NF1, NF12, and NF13 with ACE2, using MM-PBSA.

**Figure 7:**
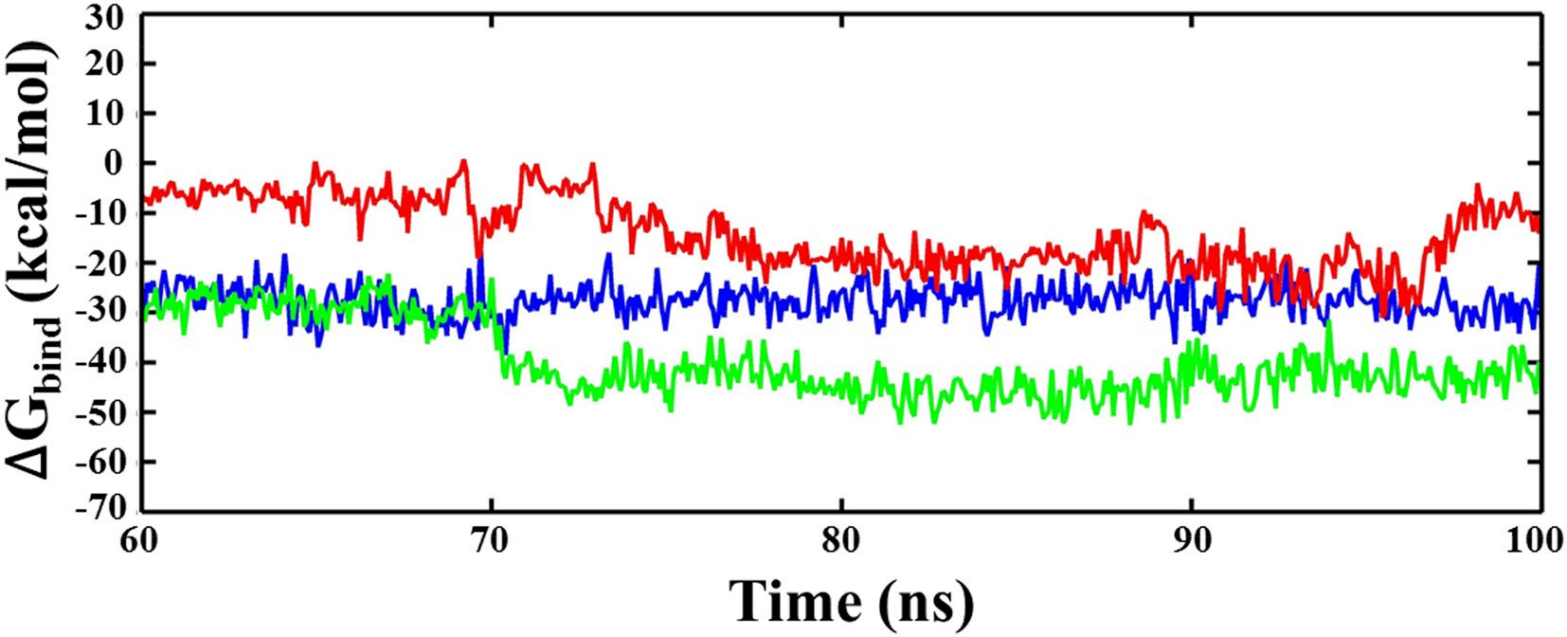
Binding free energy (ΔG_bind_) estimation of ligands, NF1, NF12, and NF13 with ACE2, using MM-GBSA.

The molecular binding stability of ligand molecules with ACE2 was also confirmed by estimating the binding free energy using MM-PBSA and MM-GBSA (Figs. 6 and 7). Both MM-PBSA and MM-GBSA comprise of various energy components, including the bonded (polar) and non-bonded (vdW: van der Waals and EEL: electrostatic), solvation free energy (ΔG_GB_), and SASA contributing for ∆G_SURF_ energetic terms to emphasize the interactions of compounds, NF1, NF12, and NF13 at the active site of ACE2. The MM-PBSA was performed on the equally spaced frames (501) taken from the last 40 ns trajectory of the protein–ligand complexes. The calculated different energy components using PB and GB models are outlined in Tables 3 and 4, respectively. Results show that all the three compounds favourably bind with the protein ACE2. The PB model shows that the binding free energy of compounds, NF1, NF12, and NF13 with ACE2 converge around at ~ 70 ns and remain consistent for the remaining period of simulation with ΔG_bind_ values: − 13.23, − 4.79, and − 20.25 kcal/mol, respectively (Fig. 6). The energy components summarized in Table 3 suggest the major contribution of van der waals energy (ΔE_VDW_) in stabilizing the ligand molecules at the active site of ACE2. The highest value of ΔE_VDW_ = − 47.13 kcal/mol obtained for NF13 and NF12 shows the lowest ΔE_VDW_ value of − 28.15 kcal/mol. The highest ΔE_EEL_ value − 7.25 to 28.15 kcal/mol was shown by NF1, and the lowest ΔE_EEL_ = − 2.84 to 28.15 kcal/mol was observed for NF12. Although the compounds NF1 and NF13 show marginal differences for the energy terms ΔE_VDW_ and EEL energies, but considering the other components polar, non-polar, and dispersion binding energies, results in the most favorable interaction of NF13 with best binding free energy (ΔG_bind_ = − 20.25 kcal/mol). NF1 shows the moderate binding affinity (ΔG_bind_ = − 13.23 kcal/mol) with ACE2 compared to NF13, whereas the least binding affinity (ΔG_bind_ = − 4.79 kcal/mol) was obtained for NF12. Figure 7 shows the binding free energy trajectory using MM-GBSA, which indicates that all three compounds, NF1, NF12, and NF13, were converged at ~ 70 ns, with ΔG_bind_ values around − 27.72, − 14.15, and − 39.92 kcal/mol, respectively. The binding energy components enumerated in Table 4. It shows similar results for the contribution of ∆E_VDW_ (NF1: − 43.13 kcal/mol, NF12: − 28.15 kcal/mol, NF13: − 47.13 kcal/mol) in stabilizing the ligands at the ACE2 active site, as observed with MM-PBSA. However, considering the solvation energy, we observed a remarkable increase in the electrostatic interaction (∆E_EEL_) interaction of compounds. Results show the highest ∆E_EEL_ value − 29.00 kcal/mol for NF1 and the lowest value of − 11.37 kcal/mol for NF12. However, the collective contribution of all defined energy components in Table 3 shows the most favorable binding of NF13 with ΔG_bind_ = − 39.92 kcal/mol, and the NF12 (ΔG_bind_ = − 14.15 kcal/mol) again shows the least affinity with ACE2. Thus, applying both models of binding free energy approximation suggests that NF13 may be explored as potential candidates for the development of ACE2 inhibitors.

**Table 3:** Binding free energy estimation of compounds using MM-PBSA.

	ΔE_VDW_	ΔE_EEL_	ΔG_POL_	ΔG_NPOL_	ΔG_DISP_	ΔG_BIND_
NF1	− 43.13 ± 2.90	− 7.25 ± 1.13	12.14 ± 1.20	− 31.41 ± 1.34	56.42 + 1.65	− 13.23 ± 2.95
NF12	− 28.15 ± 5.67	− 2.84 ± 1.73	6.23 ± 1.92	− 22.22 ± 4.39	42.19 ± 1.78	− 4.79 ± 2.21
NF13	− 47.13 ± 6.04	− 5.88 ± 1.28	8.97 ± 0.96	− 34.06 ± 3.32	57.86 ± 1.50	− 20.25 ± 3.35

**Table 4:** Binding free energy estimation of compounds using MM-GBSA.

	ΔE_VDW_	∆E_EEL_	ΔG_GB_	∆G_SURF_	ΔG_BIND_
NF1	− 43.13 ± 2.90	− 29.00 ± 4.53	49.77 ± 3.84	− 5.35 ± 0.26	− 27.72 ± 3.41
NF12	− 28.15 ± 5.67	− 11.37 ± 6.92	29.17 ± 6.21	− 3.80 ± 0.87	− 14.15 ± 6.99
NF13	− 47.13 ± 6.04	− 23.55 ± 5.12	36.60 ± 3.39	− 5.84 ± 0.59	− 39.92 ± 7.38

*All energy terms are expressed in kcal/mol.

**Figure 8:**
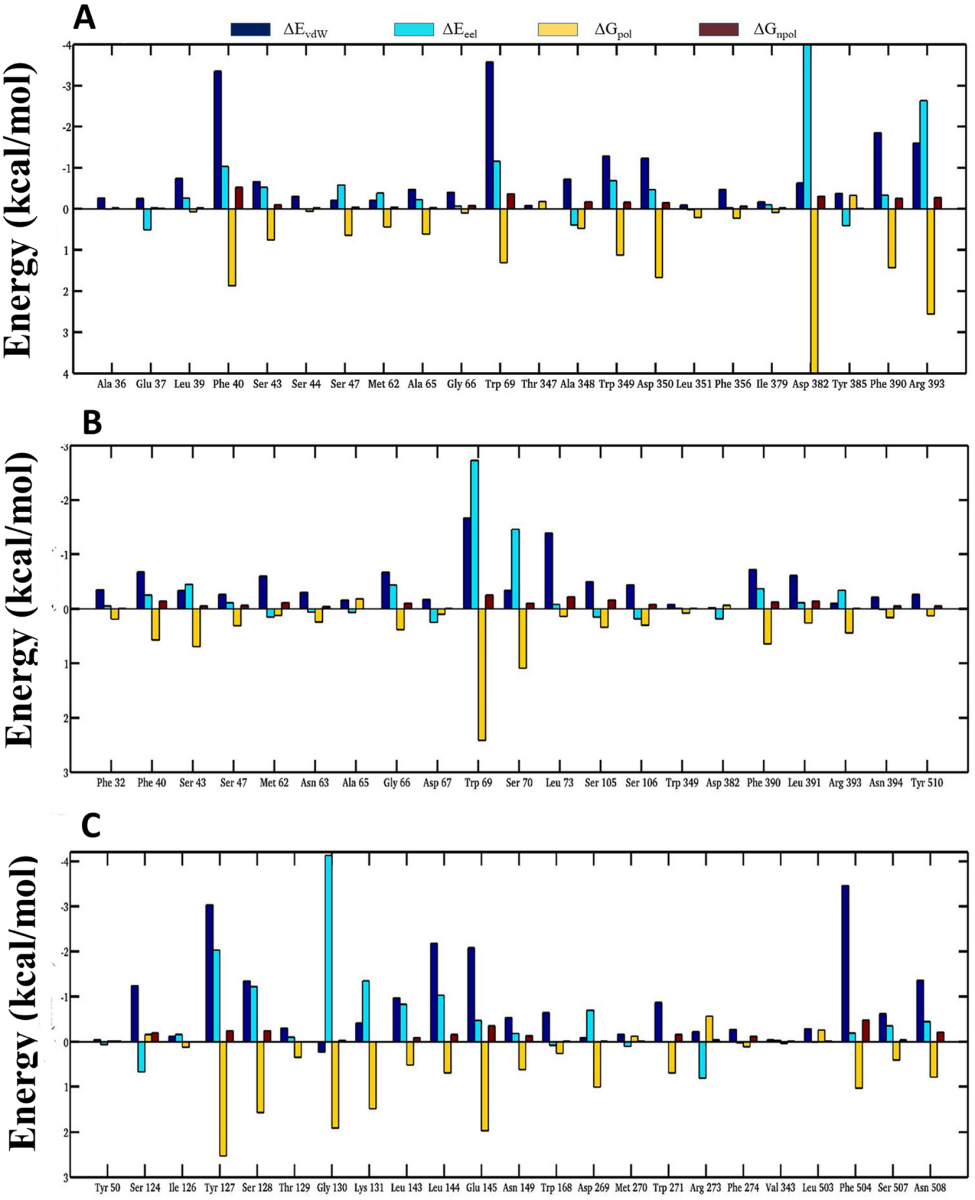
Binding energy contribution of the active site amino acids of ACE2 involve in interaction with the ligands (**A**) NF1 (**B**) NF12 and (**C**) NF13.

In order to confirm the binding progression of ligands during the simulation, we also examined the structural snapshots taken at the time interval of 10 ns (Supplementary Fig. S2). It was observed that all three ligands remain bound into the active site of ACE2 during the simulation. The spatial binding of ligands at the binding site is largely stabilized by the various molecular interactions of active site residues; thus, the contribution of binding pocket residues is crucial to quantify the binding affinity of ligands. The free energy decomposition plots ofNF1, NF12, and NF13 are shown in Fig. 8A–C, respectively. Figure 8A shows the molecular interactions of NF1 at the active site of ACE2, which is stabilized by the EEL interaction with residues Phe40, Trp69, Trp349, Asp382 and Arg393. The residues involved in vdW interactions were Leu39, Phe40, Ser43, Trp69, Ala348, Trp349, Asp350, Asp382, Phe390, and Arg393, and the non-polar interactions with Phe40, Trp69, Phe390, and Arg393, which observed consistent with the RMSF plot. The results show that the active site residues Leu39, Phe40, Ser43, Gly66, Trp69, Trp349, Asp382, Phe390, and Arg393were energetically favourable to the binding stability of ligand to the protein. Remarkably, NF1was predominantly stabilized in the binding pocket through the vdW interaction and EEL interaction, which is mostly contributed by the residues Phe40 (− 3.35 kcal/mol) and Trp69 (− 3.57 kcal/mol) and Asp382 (− 8.48 kcal/mol), and Arg393 (− 2.63 kcal/mol), respectively.

The free energy decomposition plot of NF12 shows that the binding of a ligand at the active site of ACE2 was stabilized by major contributions from vdW interactions of residues Phe40, Met62, Gly66, Trp69, Leu73, Ser106, Phe390, and Leu391 and EEL interactions with residues Ser43, Gly66, Trp69, Ser70, Phe390 and Arg393 (Fig. 8B), whereas the non-polar contribution involves the residues Phe40, Met62, Trp69, Leu73, Ser105, Phe390, and Leu391. Considering individual residues energy contribution terms, it was observed that NF12 at the active site was predominantly stabilized by the vdW interactions of TRP69 (− 1.67 kcal/mol) and LEU73 (− 1.39 kcal/mol) and EEL interactions by TRP69 (− 2.7 kcal/mol) and Ser70 (− 1.46 kcal/mol). Figure 8C shows the binding free energy of the amino acids involved in the interaction of NF13 with ACE2, which suggest the EEL contribution of Tyr127, Ser128, Gly130, Lys131, Leu143, Leu144, Glu145, Asp269, and Asn508 and, the vdW contribution of residues: Ser124, Tyr127, Ser128, Leu143, Leu144, Glu145, Asn149, Trp168, Trp271, Phe504, Ser507 and Asn508 for stabilizing NF13 at the active site of ACE2. In terms of energy contribution, the vdW was majorly noted with Ser124 (− 1.24 kcal/mol), Tyr127 (− 3.03 kcal/mol), Ser128 (− 1.34 kcal/mol), Leu144 (− 2.18 kcal/mol), Glu145 (− 2.08 kcal/mol), Phe504 (− 3.46 kcal/mol), Asn508 (− 1.36 kcal/mol) and the EEL energy with Tyr127 (− 2.03 kcal/mol), Ser128 (− 1.22 kcal/mol), Gly130 (− 4.13 kcal/mol), Lys131(− 1.35 kcal/mol), Leu144 (− 1.03 kcal/mol) contributed the highest energy. Thus, the binding free energy and energy decomposition plots suggest that the binding surface with strong vdW and EEL interactions can steadily hold the compounds in the binding pocket; also, the residues involved in non-polar interactions supported the stability of the ligands through hydrophobic interactions. Further, the molecular docking study with SARS-CoV-2 spike protein mutants (N501Y and D614G) with similar binding affinity (Supplementary Table S2 and Supplementary Fig. S3) like wild type protein with compound NF13 provided an evidence of therapeutic potential to inhibit the SARS-CoV-2 infection.

## Conclusion

In this study, we used in silico tools to extensively screen a library of triarylcouramins compounds and selected three potential triarylcouramins, NF1, NF12, and NF13, that effectively bind the interacting junction of the S-protein-ACE2 protein complex, consequently inhibiting the complex formation. The binding is stabilized by H-bonds and non-covalent interactions, as revealed by the docking studies. The protein–ligand dynamics and stability were studied by the MD simulation studies. The binding stability of ligand molecules with the protein complex was further supported by the results of binding free energy studies using MM-PBSA and MM-GBSA. The efficacy of the compounds should be further explored through in vitro and in vivo studies.

## Supplementary Information

Supplementary Information.
