# Supplementary material for: Arylcoumarin perturbs SARS-CoV-2 pathogenesis by targeting the S-protein/ACE2 interaction
Source: Sci Rep. 2022 Oct 11;12:17038. doi: 10.1038/s41598-022-20759-7 (PMC9552724; doi:10.1038/s41598-022-20759-7)
Supplement: Supplementary file 1 — Supplementary Information. [file 41598_2022_20759_MOESM1_ESM.docx]

**Arylcoumarin perturbs SARS-CoV-2 pathogenesis by targeting the S-protein/ACE2 interaction.**

Ruhar Singh^#a^, Abhijeet Kumar ^b#^, Jitendra Subhash Rane^#c^, Rajni Khan^d^, Garima Tripathi^e^, Amrendra K. Ajay^f*^, Amresh Prakash^g*^and Shashikant Ray^h*^

^a^School of Computational & Integrative Sciences, Jawaharlal Nehru University, New Delhi, India

^b^Department of Chemistry, Mahatma Gandhi Central University Motihari-845401, India

^c^Department of Biosciences & Bioengineering, Indian Institute of Technology Bombay, Mumbai -400076, India

^d^Department of Pharmacology and Toxicology, National Institute of Pharmaceutical Education and Research Hajipur-844102

^e^Department of Chemistry,T.N.B. College, Bhagalpur, Tilka Manjhi Bhagalpur University, Bhagalpur-812007, India

^f^Renal Division, Department of Medicine, Brigham and Women’s Hospital and Harvard Medical School, Boston, MA 02115, USA

^g^Amity Institute of Integrative Sciences and Health, Amity University Haryana, Gurgaon-122413, India

^h^Department of Biotechnology, Mahatma Gandhi Central University Motihari-845401, India

**^#^ These authors contributed equally to this work.**

***Correspondence may be addressed to these authors**

^f*^Amrendra K. Ajay, Renal Division, Department of Medicine, Brigham and Women’s Hospital and Harvard Medical School, Boston, MA 02115, USA, E-mail: akajay@bwh.harvard.edu

^g*^Amresh Prakash, Amity Institute of Integrative Sciences and Health, Amity University Haryana, Gurgaon-122413, India, E-mail:[amreshprakash@jnu.ac.in](mailto:amreshprakash@jnu.ac.in)

^h*^Shashikant Ray, Assistant Professor, Department of Biotechnology, Mahatma Gandhi Central University Motihari-845401, India, E-mail: [shashikantray@mgcub.ac.in](mailto:shashikantray@mgcub.ac.in)

**Keywords:** Coronavirus, ACE2, arylated coumarin derivatives, Binding site, MM/PBSA

**Running Head**: Arylcoumarin inhibited COVID-19 Pathogenesis.

**Supplementary Table 1**

| **S.No.** | **Common Name** | **Arylcoumarin** | **IUPAC Name** |
| --- | --- | --- | --- |
|  | NF1 |  | 4,6,8-tri(naphthalen-2-yl)-2*H*-chsromen-2-one |
|  | NF2 |  | 3,4,6,8-tetrakis(4-hydroxyphenyl)-2*H*-chromen-2-one |
|  | NF3 |  | 3,4,6-tris(4-hydroxyphenyl)-2*H*-chromen-2-one |
|  | NF4 |  | 4,6,8-tris(4-butoxyphenyl)-2*H*-chromen-2-one |
|  | NF5 |  | 3,4-bis(4-hydroxyphenyl)-2*H*-chromen-2-one |
|  | NF6 |  | 4,6,8-tris(4-methoxyphenyl)-2*H*-chromen-2-one |
|  | NF7 |  | 4,6-bis(3,4-dihydroxyphenyl)-2*H*-chromen-2-one |
|  | NF8 |  | 4,6,8-tris(4-hydroxyphenyl)-2*H*-chromen-2-one |
|  | NF9 |  | 4,6-bis(4-hydroxyphenyl)-2*H*-chromen-2-one |
|  | NF10 |  | 3,4,6,8-tetrakis(4-hydroxyphenyl)-2*H*-chromen-2-one |
|  | NF11 |  | 6,8-bis(4-hydroxyphenyl)-4-(naphthalen-2-yl)-2*H*-chromen-2-one |
|  | NF12 |  | 8-(4-hydroxyphenyl)-4,6-di(naphthalen-2-yl)-2*H*-chromen-2-one |
|  | NF13 |  | 8-(4-hydroxyphenyl)-3,6-di(naphthalen-2-yl)-2*H*-chromen-2-one |
|  | NF14 |  | 3-benzhydryl-4,6-dihydroxy-2*H*-chromen-2-one |
|  | NF15 |  | 6-(4-fluorophenyl)-4-(4-hydroxyphenyl)-2*H*-chromen-2-one |
|  | NF16 |  | 4,6-bis(4-isopropoxyphenyl)-2*H*-chromen-2-one |
|  | NF17 |  | 7-(diethylamino)-4-phenyl-2*H*-chromen-2-one |
|  | NF18 |  | 4,6-bis(4-hydroxyphenyl)-2-oxo-2*H*-chromene-3-carbaldehyde |
|  | NF19 |  | (E)-4,6-bis(4-hydroxyphenyl)-3-(4-hydroxystyryl)-2*H*-chromen-2-one |
|  | NF20 |  | (E)-4,6-bis(4-hydroxyphenyl)-3-(2-(naphthalen-2-yl)vinyl)-2*H*-chromen-2-one |
|  | NF21 |  | 4,6-bis(4-hydroxyphenyl)-3-(naphthalen-2-ylethynyl)-2*H*-chromen-2-one |

**Supplementary Table 1:** Structure of all the Arylcoumarin derivate used in this study

**Supplementary Table S2**

| Compounds | Docking Score (kcal/mol) | Residues Involve in Molecular Interactions |
| --- | --- | --- |
| NF1 | -8.5 | Arg355, Tyr396, Pro426, Asp428, Phe429, Thr430, Lys462, Pro463, Phe464, Glu465, Ser514, Phe515, Glu516 |
| NF12 | -6.9 | Ser375, Thr376, Lys378, Tyr380, Gly404, Asp405, Val407, Arg408, Ala411, Gln414, Val503, Gly504, Tyr508 |
| NF13 | -10.2 | Leu24, Trp64, Ile68, His66, Arg78, Lys97, Asn185, Phe186, Val213, Arg214, Ala262 |

**Supplementary Table 2:** Shows the interacting amino acid residues of SARS-CoV-2 spike protein mutants (N501Y and D614G) with compound NF1,NF12 and NF13.

**Supplementary Figures S1:**


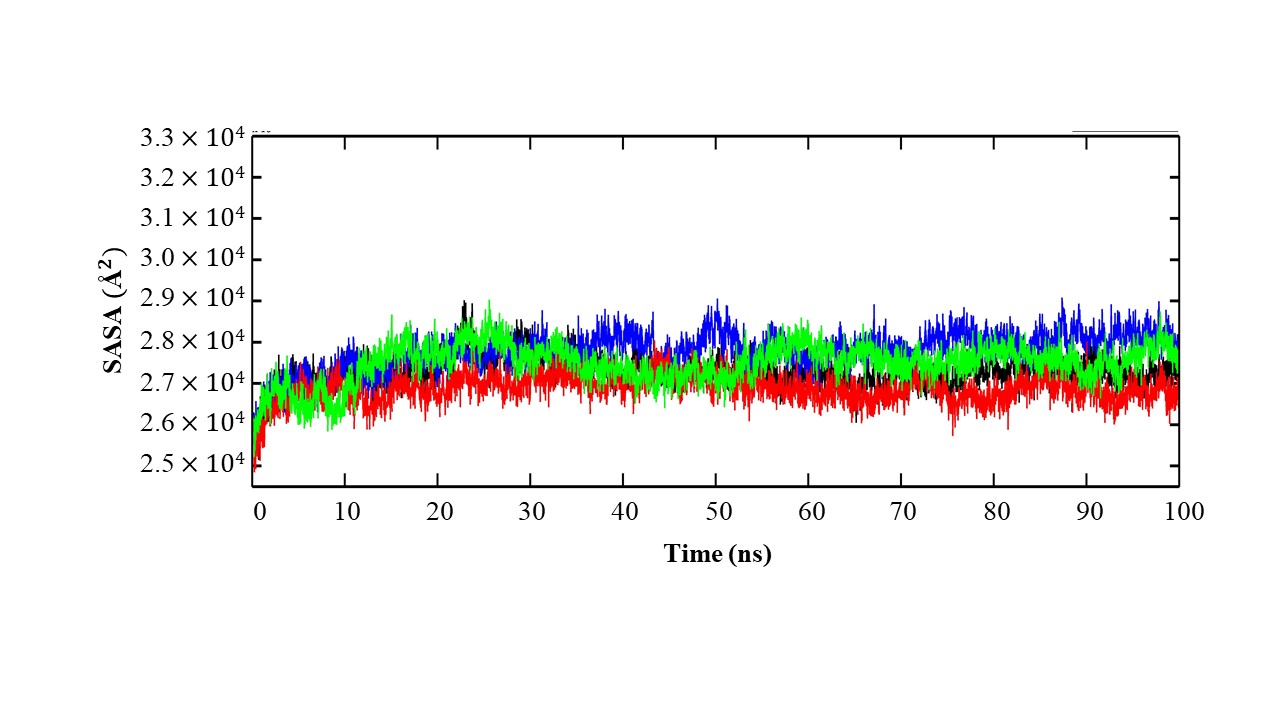


**Supplementary Figure S1.**Time evolution plot of SASA. Protein, ACE2 is shown with color black, ACE2-NF1 as blue, ACE2-NF12 with color red and ACE2-NF3 is represented by color green.


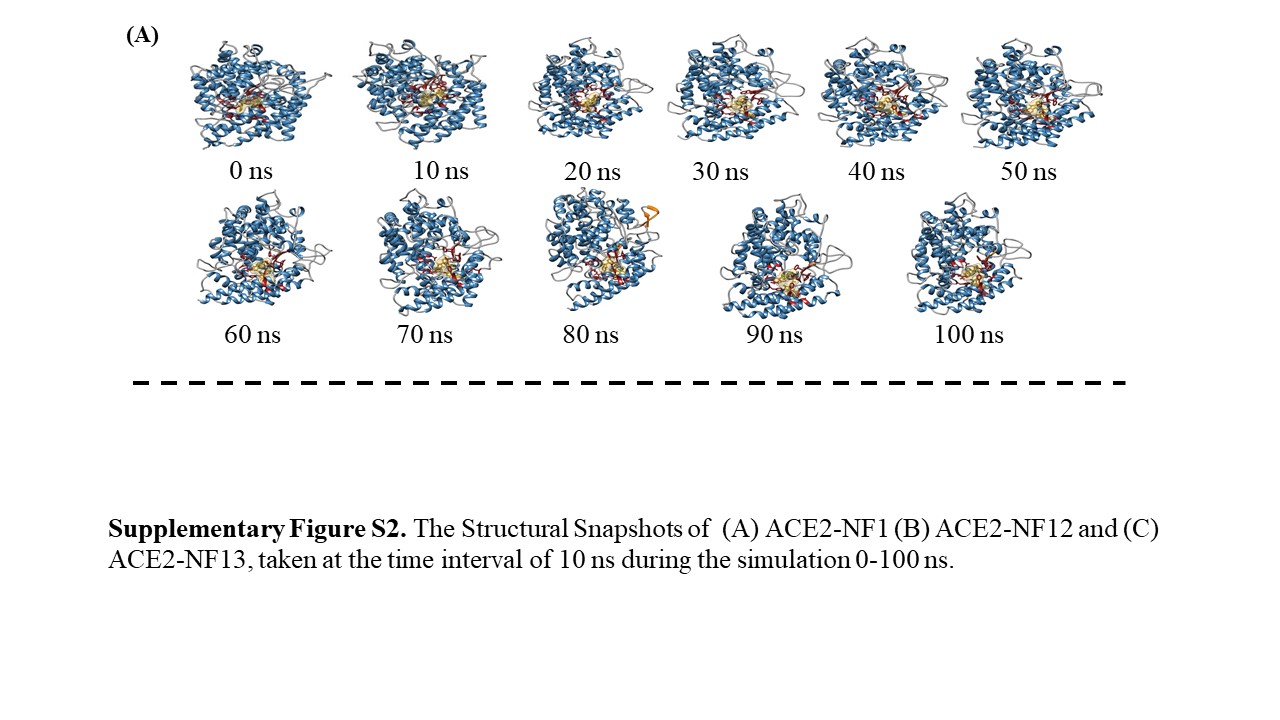


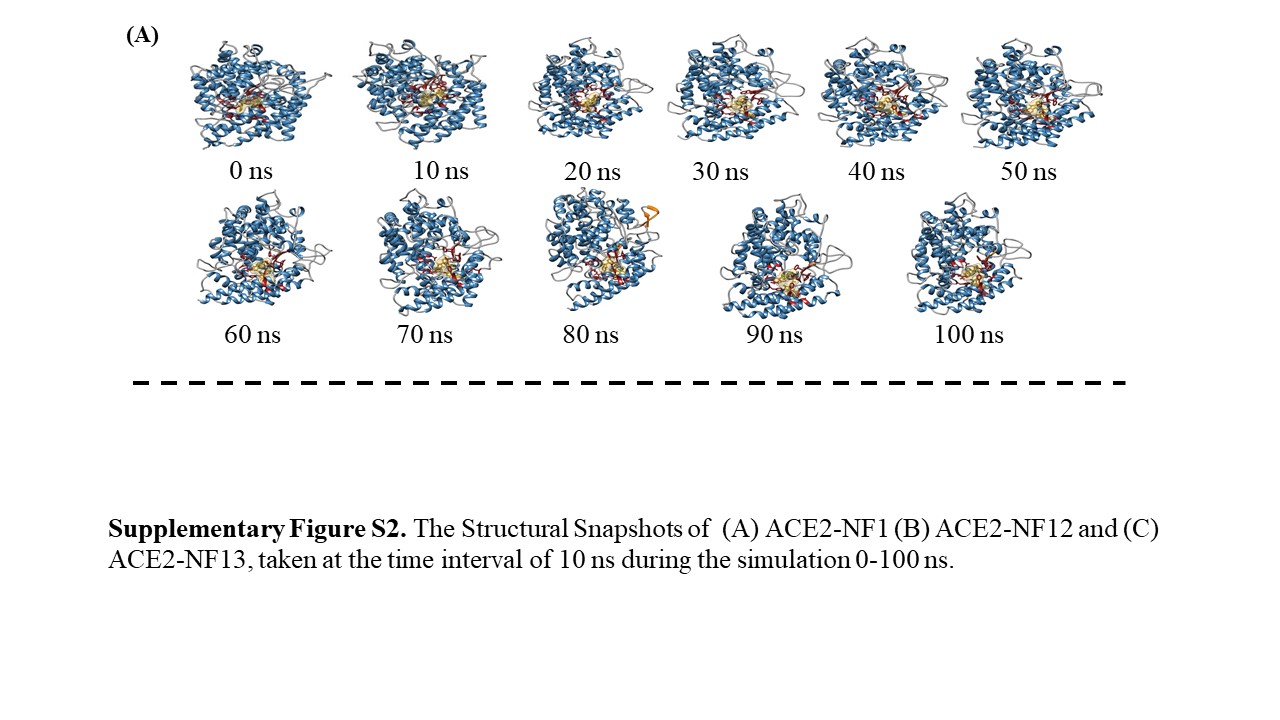

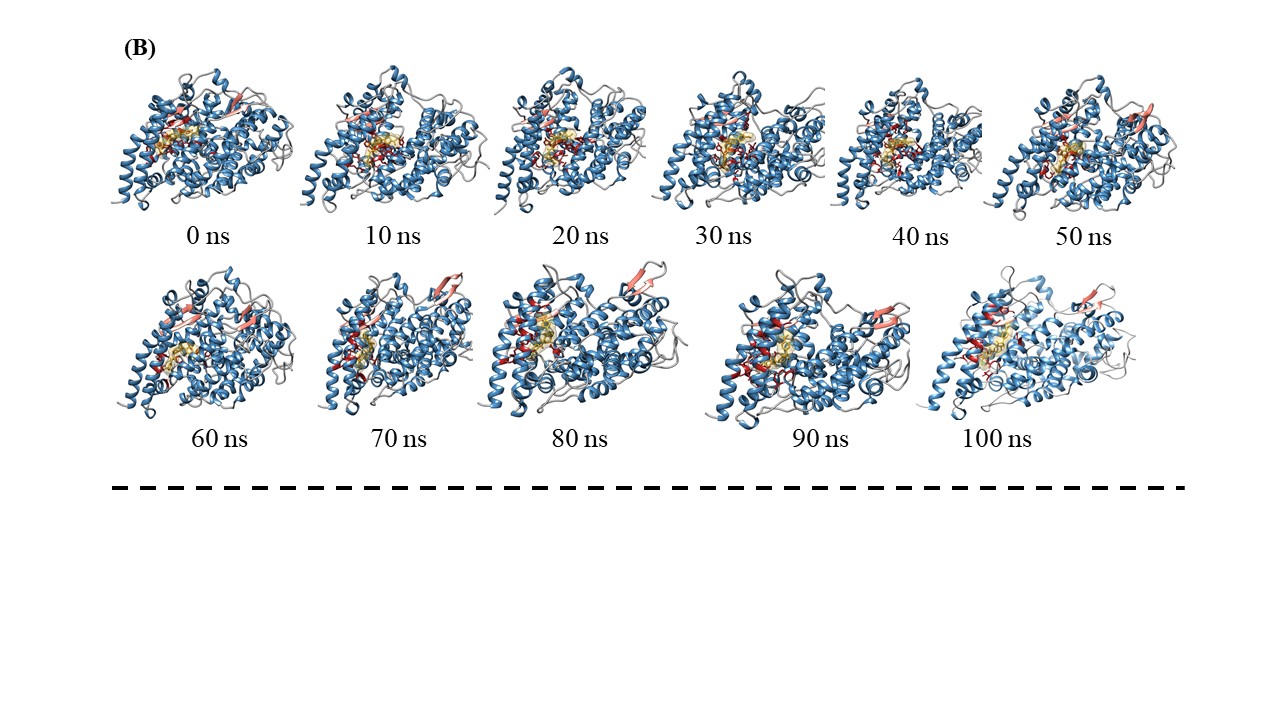


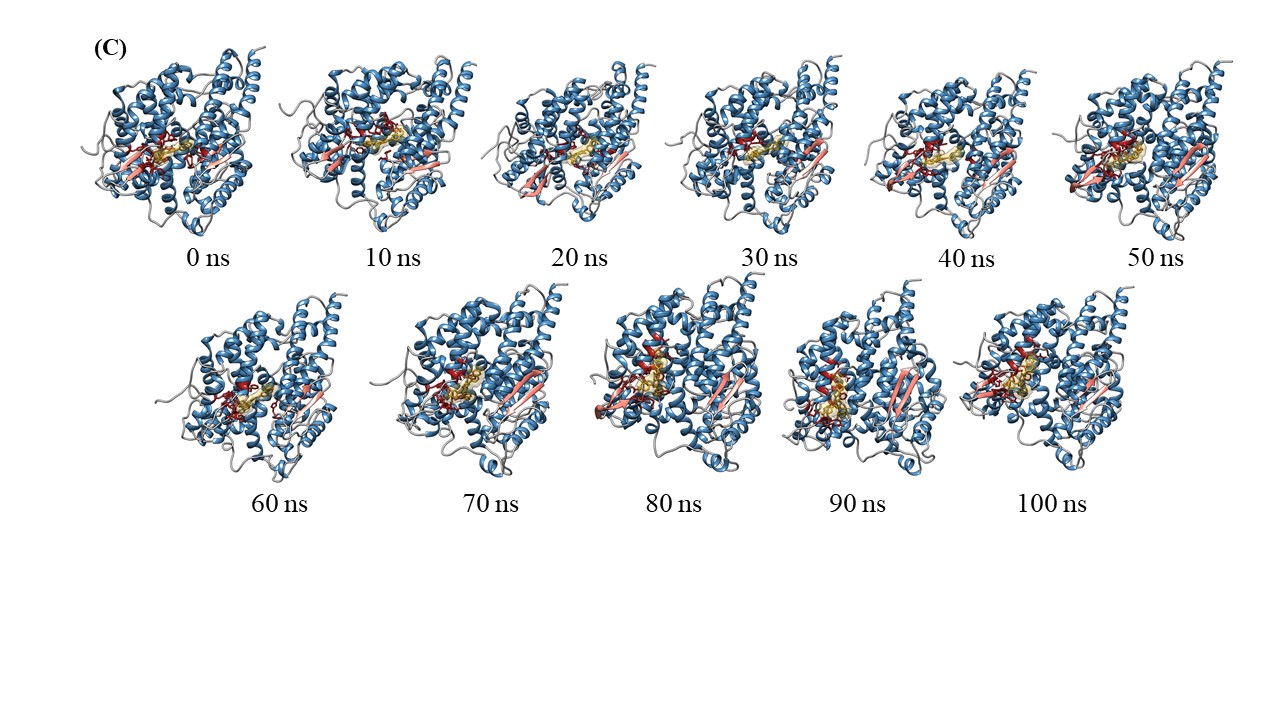


**Supplementary Figure S2.**The Structural Snapshots of (A) ACE2-NF1 (B) ACE2-NF12 and (C) ACE2-NF13, taken at the time interval of 10 ns during the simulation 0-100 ns. The protein ACE2 is shown in cartoon and the ligands, NF1, NF12 and NF13 at the active site of ACE2 are represented with stick model.

**Supplementary Figure S3:**


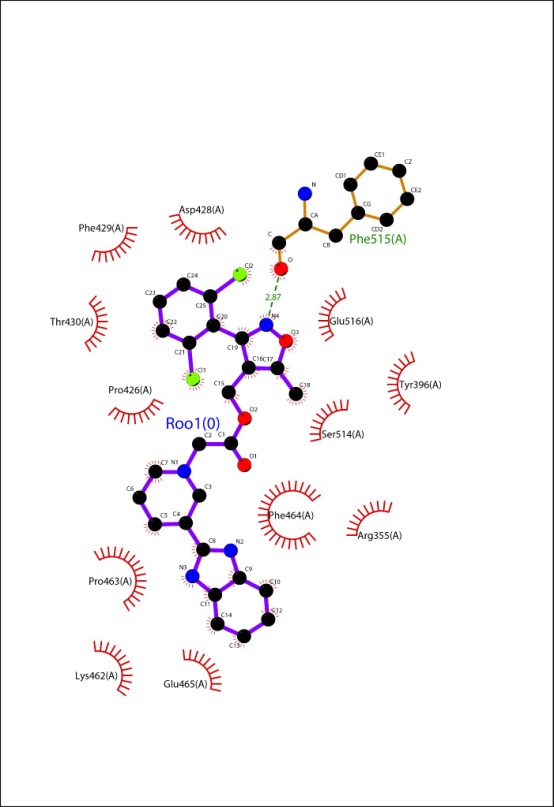

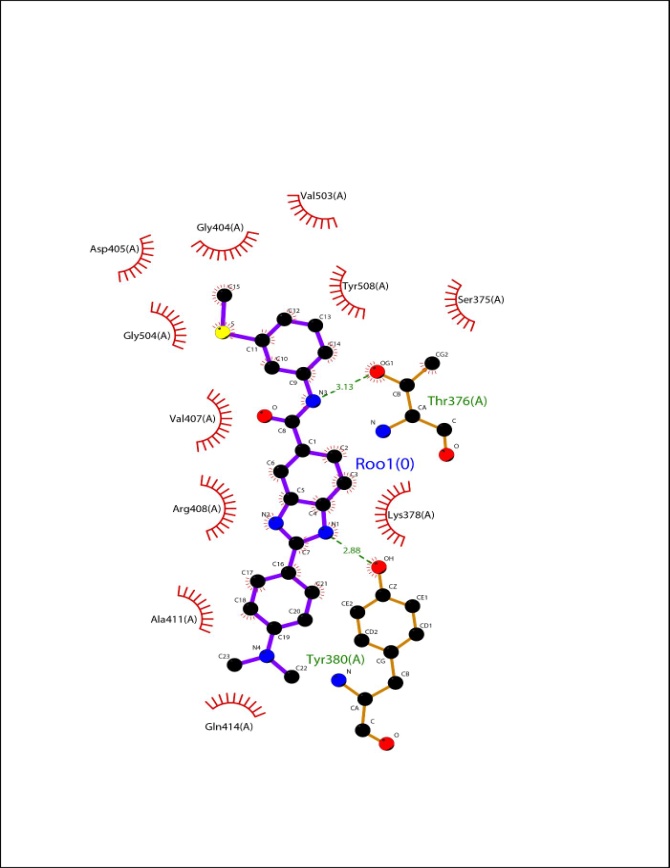

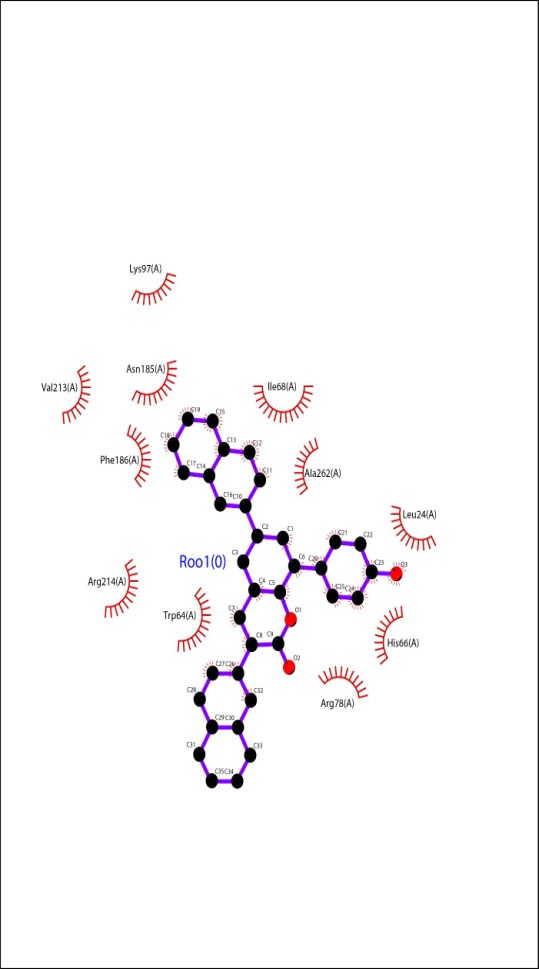


Complex with NF1 Complex with NF12 Complex with NF13

**Supplementary Figure S3:** Molecular interaction compounds (NF1, NF12 and NF13) with SARS-CoV-2 Spike protein mutants (N501Y and D614G).

**Supplementary Figure S4:**


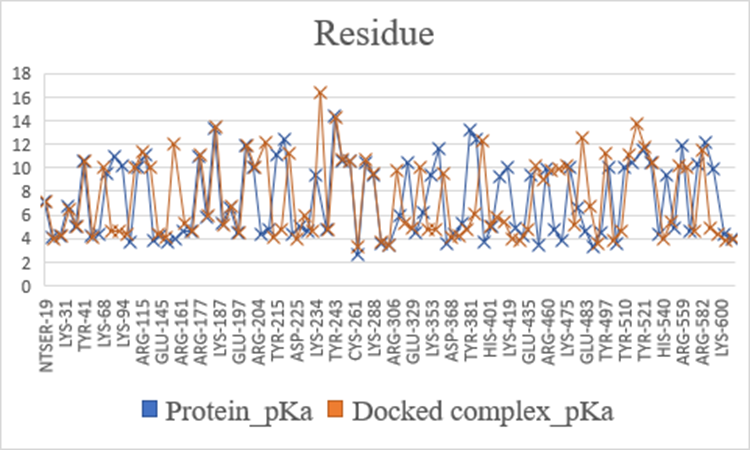


**Supplementary Figure S4**: A comparative plot of Protein and Protein-ligand docked complex. The observed values show completely overlapping values and the marginal differences at the place of decimal only.

**Supplementary Figure S5:**


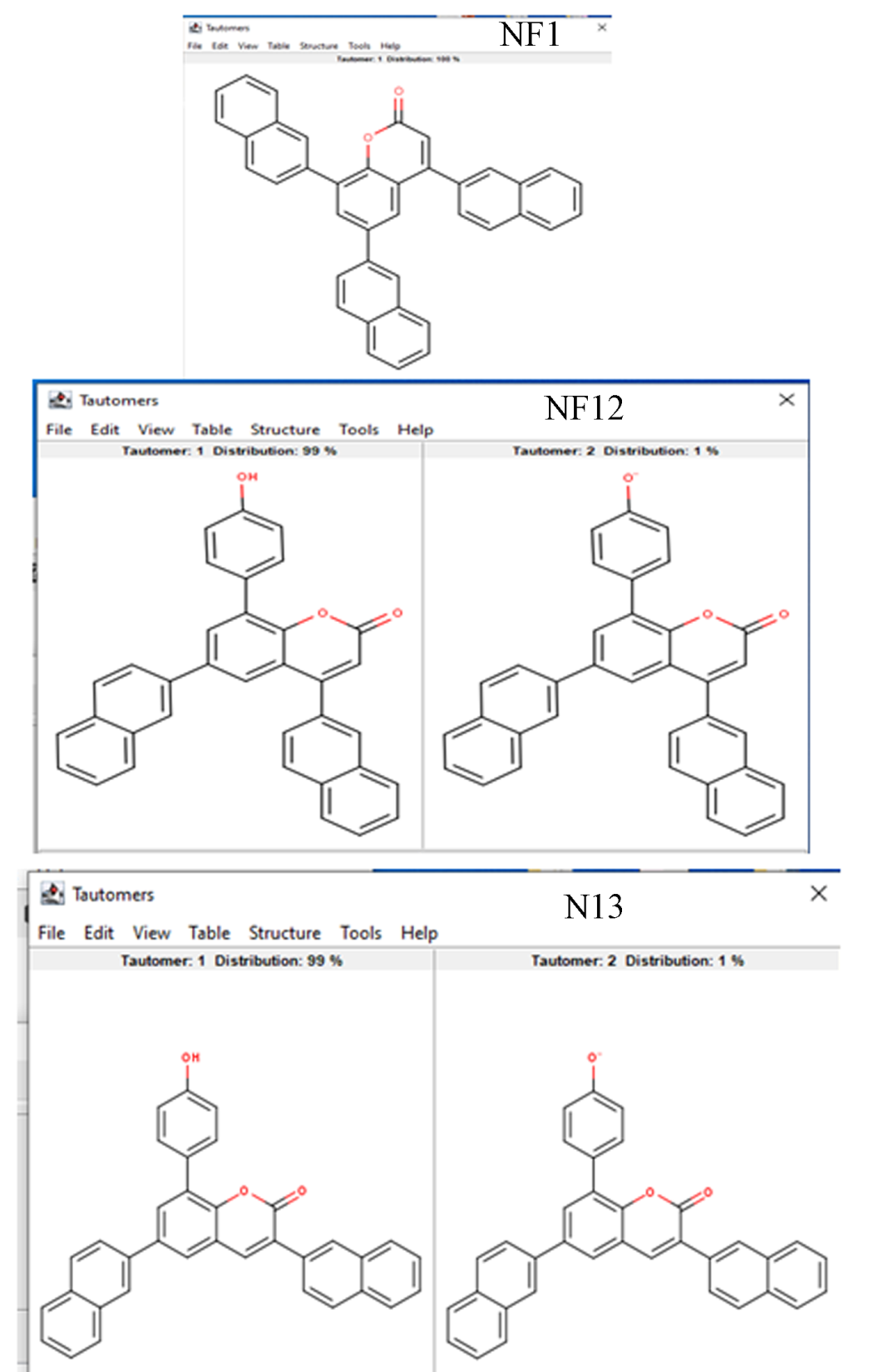


**Supplementary Figure S5***.* The protonation, tautomer, pKa, major microscopic and isoelectric point analysis using Chemaxon Marvin shows no tautomeric effect for NF1 (single tautomer 100% distribution) whereas NF12 and NF13 shows two tautomer states (docked form distributed 99%), and no isoelectric point (pI) is noted at neutral pH.
